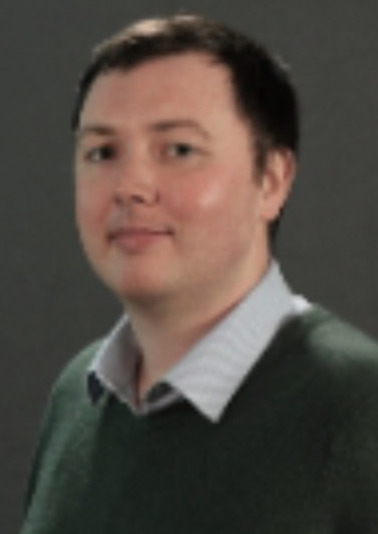# Temperature‐ and pH‐Responsive Polymeric Photocatalysts for Enhanced Control and Recovery

**DOI:** 10.1002/anie.202211132

**Published:** 2022-11-04

**Authors:** Rong Li, Katharina Landfester, Calum T. J. Ferguson

**Affiliations:** ^1^ Department School of Chemistry University of Birmingham Birmingham UK; ^2^ Max Planck Institute for Polymer Research Mainz Germany

**Keywords:** Heterogeneous Catalysis, Photocatalysis, Stimuli-Responsive Heterogeneous Catalyst, Switchable Water Compatibility

## Abstract

The emergence of heterogeneous photocatalysis has facilitated redox reactions with high efficiency, without compromising the recyclability of the photocatalyst. Recently, stimuli‐responsive heterogeneous photocatalytic materials have emerged as a powerful synthetic tool, with simple and rapid recovery, as well as an enhanced dynamic control over reactions. Stimuli‐responsive polymers are often inexpensive and easy to produce. They can be switched from an active “on” state to an inert “off” state in response to external stimuli, allowing the production of photocatalyst with adaptability, recyclability, and orthogonal control on different chemical reactions. Despite this versatility, the application of artificial smart material in the field of heterogeneous photocatalysis has not yet been maximized. In this Minireview, we will examine the recent developments of this emerging class of stimuli‐responsive heterogeneous photocatalytic systems. We will discuss the synthesis route of appending photoactive components into different triggerable systems and, in particular, the controlled activation and recovery of the materials.

## Graphical abstract

Stimuli‐responsive photocatalytic materials have emerged that are not only able to efficiently catalyze reactions but can use external stimuli to modulate their physical properties. This technique has been used to control the reactivity of a photocatalytic material turning it on/off. Moreover, photocatalytic materials have been synthesized which can be easily recycled with the use of an external trigger.

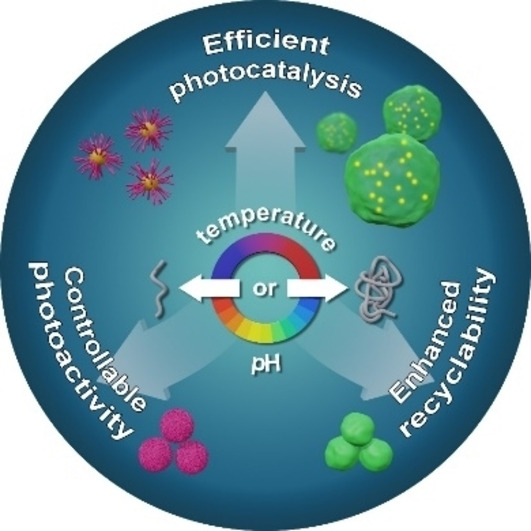

## 1 Introduction

Solar energy is a renewable abundant and eco‐friendly energy source that can be converted to chemical or electrical energies. A broad spectrum of photocatalytic materials have been developed to facilitate chemical transformations under sunlight, mimicking the natural photosynthesis process. Generally, upon visible light irradiation, an electron is promoted from the ground state to an excited state of the photocatalyst, generating a hole in the ground state and an electron in the higher energy excited state. This excited electron–hole pair can be utilized directly for either reduction or oxidation reactions, respectively. To date, photocatalytic systems have been employed in a large range of applications including water pollutant degradation,[^1^, ^2^, ^3^, ^4^] antibacterial treatment,[^5^, ^6^, ^7^, ^8^] photodynamic therapy,[^9^, ^10^, ^11^, ^12^] hydrogen evolution,[^13^, ^14^, ^15^, ^16^, ^17^] carbon dioxide reduction,[^18^, ^19^, ^20^, ^21^] and organic transformations.[^22^, ^23^, ^24^, ^25^, ^26^, ^27^]

Photocatalysts are conventionally categorized as either homogeneous or heterogeneous. Homogeneous small molecular photocatalysts generally exhibit very high performance and good solubility in a selected reaction medium. However, these materials suffer from a number of inherent drawbacks in terms of difficulties in separation and recycling from the reaction mixture, which increases the production cost and pollution. Moreover, homogeneous systems are often less stable and degrade over time because of photobleaching or solvolysis.[^28^, ^29^, ^30^] Consequently, heterogeneous photocatalysts have emerged as an effective and promising alternative to homogeneous photocatalysts. Therefore, the formation of a heterogeneous photocatalyst by either immobilizing homogeneous small molecular photocatalysts to a secondary species or incorporating the photoactive species into a conjugated network is of great interest that can in principle facilitate their recovery and recycling. Many different types of materials have been utilized as support materials, including inorganic clays,^[31]^ silica gels,^[32]^ and zeolites;^[33]^ carbon‐based materials, e.g. graphene,[^34^, ^35^] C_3_N_4_,^[36]^ and carbon nanotubes,^[37]^ as well as polymeric materials such as linear or cross‐linked polymer chains, polymer particles or gels, dendrimers,^[38]^ and conjugated porous materials.^[39]^ A broad spectrum of heterogeneous photocatalysts based on classic polymers have also been summarized and discussed in one of our recent reviews.^[40]^

Among these materials, pseudo‐homogeneous polymeric photocatalysts are particularly attractive as they are able to facilitate efficient photocatalytic reactions in a homogeneous manner, where the polymers are fully solvated/swollen and the photoactive centers are easily accessible towards mass diffusion and light penetration. However, one of the critical challenges within pseudo‐homogeneous photocatalysts is the requirement of a rapid separation and efficient recycling of the catalyst, in order to minimize the production costs, reduce waste generation (e.g. organic solvents for product extraction or polymer precipitation), and diminish the metal contamination (in terms of metal‐based photocatalysts) in final products. To achieve this, photocatalytic materials can be designed with a switchable solubility or dispersibility in response to external stimuli. Ideally, the photocatalytic species are in a swelling state, where the photocatalytic centers are readily accessible, resulting in efficient catalysis, whereas upon the application of an external stimulus the catalytic systems could undergo a conformational change, leading to a phase separation and thereafter, easy recycling. Moreover, altering the solubility of the materials results in a controlled activation and deactivation of the catalyst by regulating the accessibility to the catalytic sites. This feature of orthogonally switchable functions or activities is typical of biological systems that can modulate their activity dynamically by external triggers.

As an example, enzymes can alter their internal structure/conformation and, therefore, functionalities upon the application of inhibitors,[^41^, ^42^] cofactors,^[43]^ and environmental changes (i.e. temperature,^[44]^ pH,[^45^, ^46^, ^47^] light,^[48]^ and ionic strength^[49]^). Taking lessons from nature's catalysts, different types of artificial smart materials have been developed with tunable physical properties in response to external stimuli, including light, temperature, pH value, salts, magnetic field, and mechanical force. A wide range of variations in physical properties can be observed, for instance changes in solubility, shape, volume, color, aggregation state, magnetic properties, and mechanical properties. The emergence of stimuli‐responsive photocatalytic systems has fully taken the advantages of the dynamic behavior of smart materials. It is possible to controllably switch the photocatalyst from an active “on” state to an inert “off” state, providing the opportunity to manipulate photocatalytic reactions temporally, spatially, and orthogonally to other processes.

In this Minireview, some of the latest investigations on polymer/oligomer‐based triggerable and switchable photocatalytic systems in the field of heterogeneous catalysis are discussed. Photocatalytic reactions involved in these systems have been summarized in Table 1. Considering the complexity of photocatalytic reactions and conditions involved in previous works, the comparison of photocatalysis efficiency and the analysis of detailed chemical mechanisms regarding catalytic reactions are out of the scope of this review. In the following, the progress in two types of stimuli‐responsive heterogeneous photocatalysis (temperature and pH) will be discussed in terms of the recycling of the materials and/or the dynamic control on the photocatalytic activity.

**Table 1:** Photocatalytic reactions undertaken with temperature/pH‐responsive photocatalytic polymers separated by photoactive and stimuli‐responsive moieties.

Entry	Photocatalytic moiety	Stimulus‐responsive moiety	Stimulus	Reactions	Ref.
**1**	BP	NIPAM	Temperature	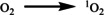	[52]
**2**	EY	NIPAM	Temperature	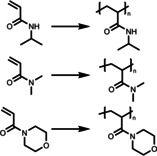	[55]
**3**	Zn‐porphyrin	NIPAM	Temperature	Rhodamine/methylene blue degradation	[56, 57, 58]
**4**	PTH	NIPAM	Temperature	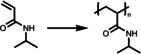	[59]
**5**	PhBTPH	NIPAM	Temperature	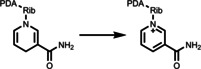	[50]
**6**	Ru(bpy)_3_Cl_2_	PIB	Temperature	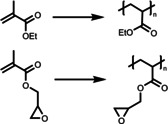	[61]
**7**	*fac*‐Ir(ppy)_3_	PIB	Temperature	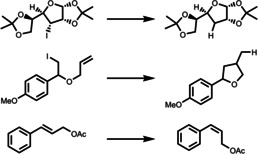	[62]
**8**	*fac*‐Ir(hdppy)_3_	C_17_H_35_‐	Temperature	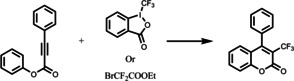	[63]
**9**	CMP	Azulene units	pH	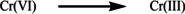	[78]
**10**	Conjugated polymer	Tertiary amine terminated alkyl chain	pH	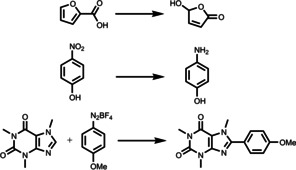	[79]
**11**	Fe‐TPP	Histidine‐PLA	pH	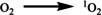	[80]
**12**	PDIPAEMA	PDIPAEMA	pH	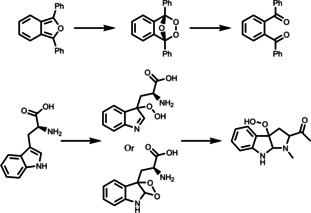	[81]
**13**	PDMAEMA	PDMAEMA	pH	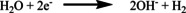	[51]
**14**	PMAA	PMAA	pH	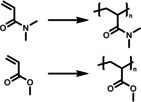	[82]

## 2 Thermo‐Responsive Polymer Photocatalytic Systems

Temperature is the most widely used physical stimulus in smart materials. A great number of thermo‐responsive classical polymers are featured by either upper critical solution temperature (UCST) or lower critical solution temperature (LCST).Typically, manyapplications of thermo‐responsive systems are based on LCST poly(*N*‐isopropyl acrylamide) (PNIPAM), because of its easy production and transition temperature (ca. 32 °C) in the range of body temperature. At temperatures below LCST, PNIPAM is hydrophilic and fully solvated in water, whereas at temperature above LCST, PNIPAM polymers become hydrophobic and undergo a phase transition forming globules or aggregations. This temperature‐induced phase transition has been exploited in the field of heterogeneous photocatalysts to enable in situ control of the photocatalysis and a number of thermo‐responsive photocatalysts based on PNIPAM polymers have been produced. Here, a high catalytic performance is observed when the photocatalytic centers are homogeneously solvated/distributed in the reaction medium in a non‐aggregate state of the material, whilst a suppressed reactivity is obtained after the formation of aggregates. In the pseudo homogeneous state, the accessibility of reactants and light to the active catalytic sites is higher in comparison to the aggregated state.[^50^, ^51^] However, the aggregation is a continuous process and heat can also be supplied as an extra source of energy in addition to light. Further to switching the hydrophilicity of the polymer systems, it can in principle accelerate the diffusion of chemical species. As a result, an acceleration in the reaction rate may also be seen in the loose aggregation state that creates a confined preferential microenvironment for the reactive species.^[52]^ In most cases, these two effects are combined, resulting in switchable photocatalysis modulated by switchable accessibility of the active catalytic centers. Aggregates formed through physical interactions, e.g. π–π stacking^[53]^ and amphiphilic self‐assembly,^[54]^ have also been utilized to induce enhanced photocatalysis, because of the aggregation‐induced triplet excited state generation or charge carrier migration, which is not discussed in this review. Furthermore, the phase transition from hydrophilic to hydrophobic could induce a phase separation of the polymers that can be used for the recovery of the photocatalytic polymer.

### 2.1 Switchable Chain Solubility

**Figure 1:**
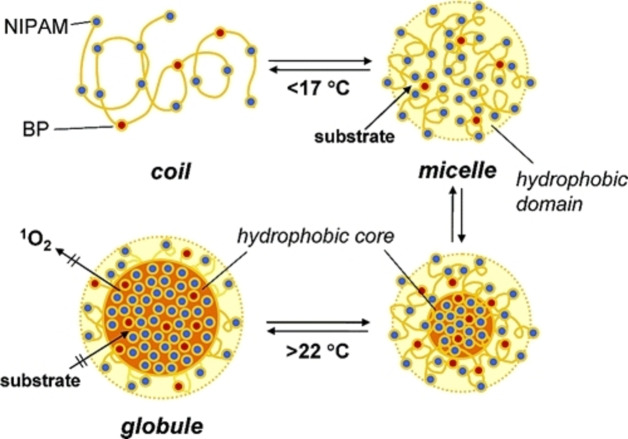
Structure of poly(NIPAM‐*co*‐BP) and changes in structure of poly(NIPAM‐*co*‐BP) in water as a function of temperature. Reproduced with permission from ref. [52].

Linear polymers are the simplest form of a polymer; they can be dissolved either completely creating a homogeneous system, or dispersed in a heterogeneous system. Koizumi *et al*.^[52]^ used PNIPAM to produce a temperature‐controlled photooxygenation polymer containing benzophenone as the photocatalytic molecule (Figure 1). This photocatalytic polymer was simply synthesized by radical copolymerization of NIPAM and 4‐allyloxybenzophenone (BP). The photooxygenation of the material exhibited a heat‐induced enhancement below 17 °C and suppression above 22 °C. This off‐on‐off behavior of poly(NIPAM‐*co*‐BP) was driven by a temperature‐induced conformational change of the polymer from coil to micelle to globule state. When the temperature was in the range of 5–17 °C, loose aggregations of poly(NIPAM‐*co*‐BP) were formed. This micelle state was capable of extending the lifetime of the ^1^O_2_ because of the increased hydrophobic domain, leading to an enhancement of photooxygenation. It is also possible to expect an additional effect, resulting from the increased diffusion rate of the mass transportation that is triggered by the elevated temperature in the loose aggregation state. With further increased temperature above 22 °C, the polymer transforms from a micellar state to a globule state containing a hydrophobic precipitated core that blocks access to photoactive sites, resulting in a suppression in photoactivity. A slightly lower LCST temperature (29 °C) was observed compared to pure PNIPAM polymer chains (32 °C), which is possibly due to the increased hydrophobicity from the BP units (6 mol %). It suggests that LCST can be tuned according to the interest of application by adjusting the doping of BP units. After use, this material can be easily recovered by heating up to 40 °C, followed by centrifugation. These features suggest the combination of PNIPAM and small photocatalyst molecules is a simple and effective approach for the production of smart photocatalysts. As a preliminary investigation of combining small molecule photocatalyst with stimuli‐responsive polymers, authors have provided insights by taking the advantage of the macromolecular structure change to achieve a dynamic control over the photocatalytic reaction and the recycling of the material.

Apart from the aforementioned simple photooxygenation, the application of tagging organic small molecule photocatalyst onto NIPAM polymer chains has been further investigated by Sumerlin and co‐workers.^[55]^ Here, an eosin Y (EY)‐derived NIPAM polymer photocatalyst was demonstrated for the synthesis of polymeric bioconjugates. EY was modified with an acrylate functional group, enabling the copolymerization with NIPAM monomers via RAFT polymerization. This copolymer photocatalyst was utilized for grafting *N*,*N*‐dimethylacrylamide (DMA) and 4‐acryloylmorpholine (NMO) polymers from a protein‐modified macroCTA via photoelectron/energy transfer reversible addition–fragmentation chain transfer (PET‐RAFT) polymerization. A comparable efficiency as the unmodified EY was obtained using poly(EY‐NIPAM). Post polymerization, the photocatalyst was removed by heating the reaction mixture to 37 °C and filtrating. This straightforward photocatalyst removal protocol allows small volume reactions and significantly lowers the cost of purification. Moreover, the polymerization can be performed in the presence of a protein in biologically benign conditions, demonstrating its usefulness as a biocompatible photocatalyst for advanced bioconjugate manufacturing.

**Figure 2:**
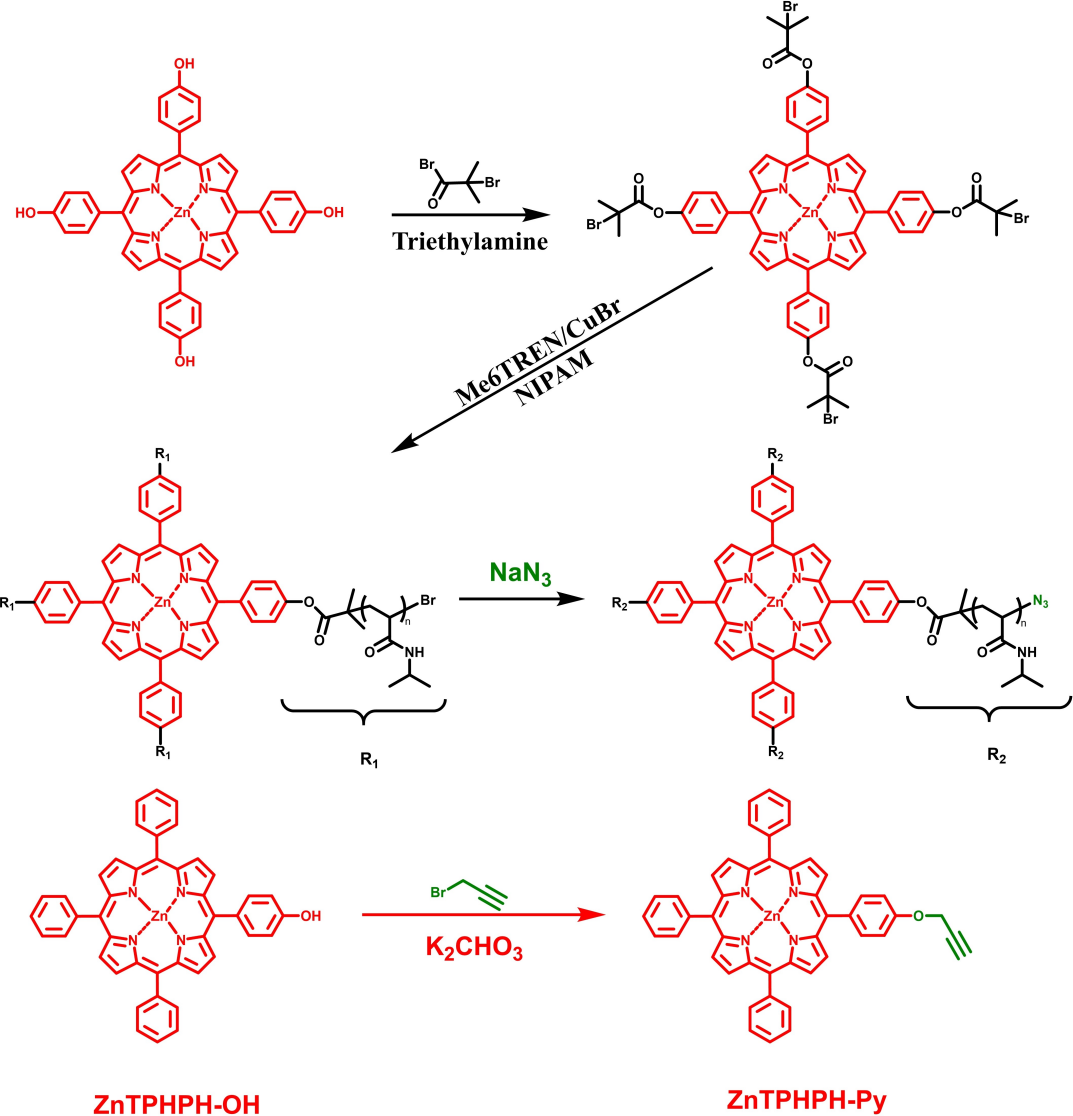
Synthesis routes of ZnTHPP‐(PNIPAM‐N_3_)_4_ and ZnTPP−Py. Reproduced with permission from ref. [58].

In addition to pure organic photocatalyst, several Zn‐based porphyrin temperature‐sensitive photocatalytic systems[^56^, ^57^, ^58^] have been reported. Generally, the central porphyrin rings were easily functionalized with four initiators for the chain extension of NIPAM via atom transfer radical polymerization (ATRP), forming star‐shaped polymers. These photocatalytic materials exhibited a LCST behavior and their temperature‐dependent dye degradation was investigated. These materials could be recovered and reused over 8 cycles with negligible loss of reactivity. The recovery was achieved by simply increasing the temperature, resulting in a heterogeneous separation of the photocatalyst. Moreover, the central porphyrin can also be further modified by different functional end groups, for example azide (N_3_) and alkyne that can enable click reactions (Figure 2).^[58]^ This method involved a two‐step reaction: the formation of NIPAM arms through ATRP and followed with the azide–yne click reaction between two photocatalytic units. However, the investigations of these photocatalytic materials have been limited to dye degradation reactions.

### 2.2 Gel Swelling and Contraction

Stimuli‐responsive cross‐linked gel systems can be synthesized in a similar manner to linear polymers, but in the presence of an appropriate cross‐linker. These network systems are generally more robust and can be recovered more easily. Moreover, because of the possibility of controlled swelling and shrinkage, they have been targeted as an effective class of smart heterogeneous photocatalysts for controllable photocatalysis.

Recently, Chen *et al*.^[59]^ synthesized PNIPAM gels containing 10‐phenylphenothiazine (PTH) photocatalyst units that were functionalized with an acrylamide group. PNIPAM was selected because of the good solubility in many organic solvents, as well as its LCST behavior in water. The Gel‐PTH was synthesized by radical polymerization of NIPAM and PTHMA monomers with the addition of *N*,*N*′‐methylenebis(acrylamide) (MBAA) as the cross‐linker. The swelling behavior of the gels was characterized by storage moduli (*G*′), ensuring a strong and stable structure for easy handling and removal. In addition to light, this gel material can also be switched ON/OFF by using temperature as a second external trigger. Here, upon elevating the temperature a reduction in the gel size as well as a transparent‐to‐opaque transition was observed. This decreased accessibility of the active photocatalytic sites within the material in response to heat, allowing the control of the reactivity. The authors employed this material to selectively catalyze the radical‐controlled polymerization (RCP) of a variety of monomers, including acrylamide, acrylate, methacrylate, vinyl ester and vinyl amide. The polymer chain propagation can be stopped and restored by tuning the temperature of the reaction medium. Here, temperature acts as a trigger to obtain an orthogonal and spatial control of the polymerization procedure. As the gel is around 1 centimeter in size, it can be removed easily and efficiently reused over six cycles without diminished performance in polymerization rate.

**Figure 3:**
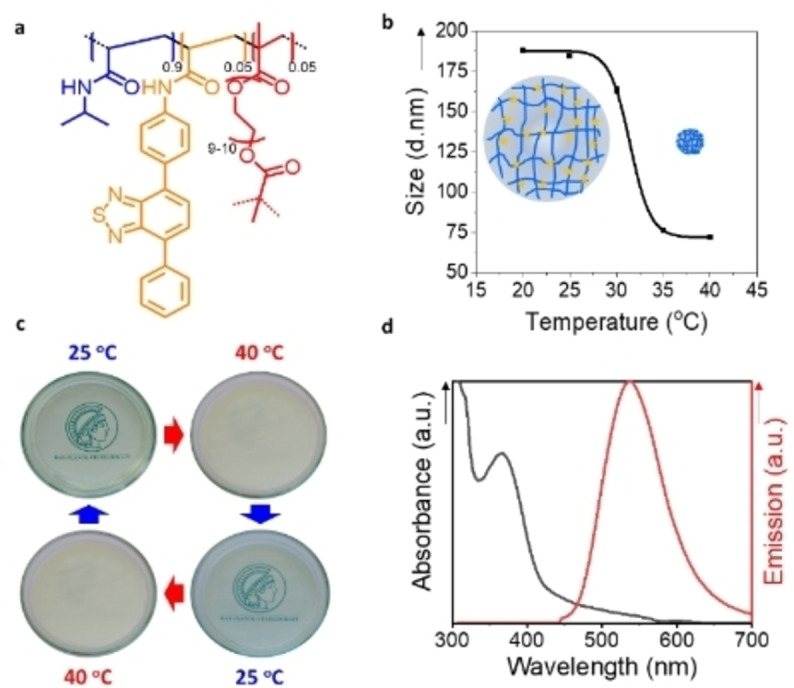
Photocatalytic temperature‐responsive microgels. a) Structure of cross‐linked photocatalytic microgel. b) Temperature‐dependent shrinking of microgel. c) Controllable optical properties of photocatalytic microgel as a function of temperature. d) UV/Vis absorption and photoluminescence spectra of the photocatalytic microgel. Reproduced with permission from ref. [50].

The dimension and production of thermo‐responsive photocatalyst gels was further extended by Ferguson *et al*. by producing NIPAM microgels containing a *N*‐(4‐(7‐phenylbenzo[*c*][1,2,5]thiadiazol4‐yl)phenyl) (PhBTPH) moiety as photocatalyst (Figure 3).^[50]^ Here, PhBTPH was functionalized with an acrylamide group that roughly matched with the propagation rate of NIPAM monomer, enabling a homogeneous distribution of the photocatalytic units across the microgel network. The authors synthesized dual‐responsive microgels through a free‐radical precipitation polymerization technique. The size of these microgels experienced a significant decrease after the application of heat, leading to the inaccessibility of the active photocatalytic sites implemented in the network. In detail, photocatalytic PNIPAM microgels were fully solvated in aqueous solution at room temperature (25 °C), enabling efficient pseudo‐homogeneous photocatalysis where the photocatalytic PhBTPH units were easily accessible towards substrate diffusion as well as light penetration. Upon heating up to 40 °C, the microgels were turning hydrophobic and phase separated from the aqueous solution, which strongly scattered light as illustrated by a significant change in the optical properties of the microgels in Figure 3c. Meanwhile, this phase separation led to a constraint mass diffusion to the photoactive centers.

Taking advantage of this alternation in macromolecular structure of photocatalytic microgels triggered by heat, the authors were able to switch on/off the photocatalytic materials. The controlled activation of these gels triggered by temperature was investigated by the selective photooxidation of the enzyme cofactor NADH. The dynamic behavior of this bioorthogonal material provided a precise control over the reaction, where the reaction can proceed at low temperature, otherwise can be stopped upon heating up. Additionally, these microgels were further used for other redox reactions, including disulfide formation mimicking the enzyme‐controlled post‐translational peptide modifications undertaken in cells. In principle, the conformational change and aggregation induced by heat can be used for the recycling of the microgel. The recovery and reusability of these gels should also be taken into consideration for further investigations.

### 2.3 Thermomorphic Systems

Further to PNIPAM‐based photocatalytic systems, a number of thermomorphic phase‐selective systems (TMSs) coupled with iridium‐ and ruthenium‐based photocatalysts have been developed. TMSs consist of at least two solvents featured with a highly temperature‐dependent miscibility gap, exhibiting two liquid phases at lower temperature and one single phase at elevated temperature. Under reaction conditions, substrates and catalyst are solvated in one single liquid phase, enabling homogeneous photocatalysis, whilst under separation conditions, products and catalyst are separated into two different liquid phases.^[60]^ A number of thermomorphic phase‐selective photocatalysts have been produced by attaching polyisobutylene (PIB) to the ligands of the metal complexes that have a strong preference for one solvent in a biphasic system, facilitating efficient photocatalysis and easy recycling of photocatalyst.

Priyadarshani *et al*.^[61]^ demonstrated a PIB‐functionalized Ru(bpy)_3_PF_6_ complex system, utilized as a soluble recyclable photoredox catalyst. Here, PIB (*M*_n_=2.3 kDa) was initially functionalized with a halogen atom prior to be grafted to the bipyridine ligands in the presence of an alkylating agent, affording at least one PIB chain per bipyridine ligand. After coupling with PIB, these ligands coordinated with metal ions. This complex photocatalyst was utilized for the free radical polymerization of acrylate monomers in heptane, a good solvent for both the photocatalyst and acrylate monomers. Upon the extension of the polymer chains, polyacrylate polymers become insoluble in heptane and self‐separate from the photocatalyst with negligible Ru contamination. This significant reduction in Ru leaching, compared to the unmodified Ru(bpy)_3_PF_6_ photocatalyst, is due to the high affinity of the PIBs for the solvent. The photocatalyst complexes can be recycled for at least three times obtaining polymer yields greater than 70 %. Moreover, the PIB−Ru catalyst system that consists of 2 PIB per bipyridine ligand displayed an additional phase‐selective solubility in a thermomorphic heptane/DMF system. One single phase of the photocatalyst was obtained in heptane/DMF mixture at high temperature, while upon cooling a phase separation occurred resulting in a biphasic system separating photocatalyst from DMF. DMF, as a very powerful solvent for a variety of reactions, can effectively expand the application of this homogeneous photocatalyst with simple recyclability.

**Figure 4:**
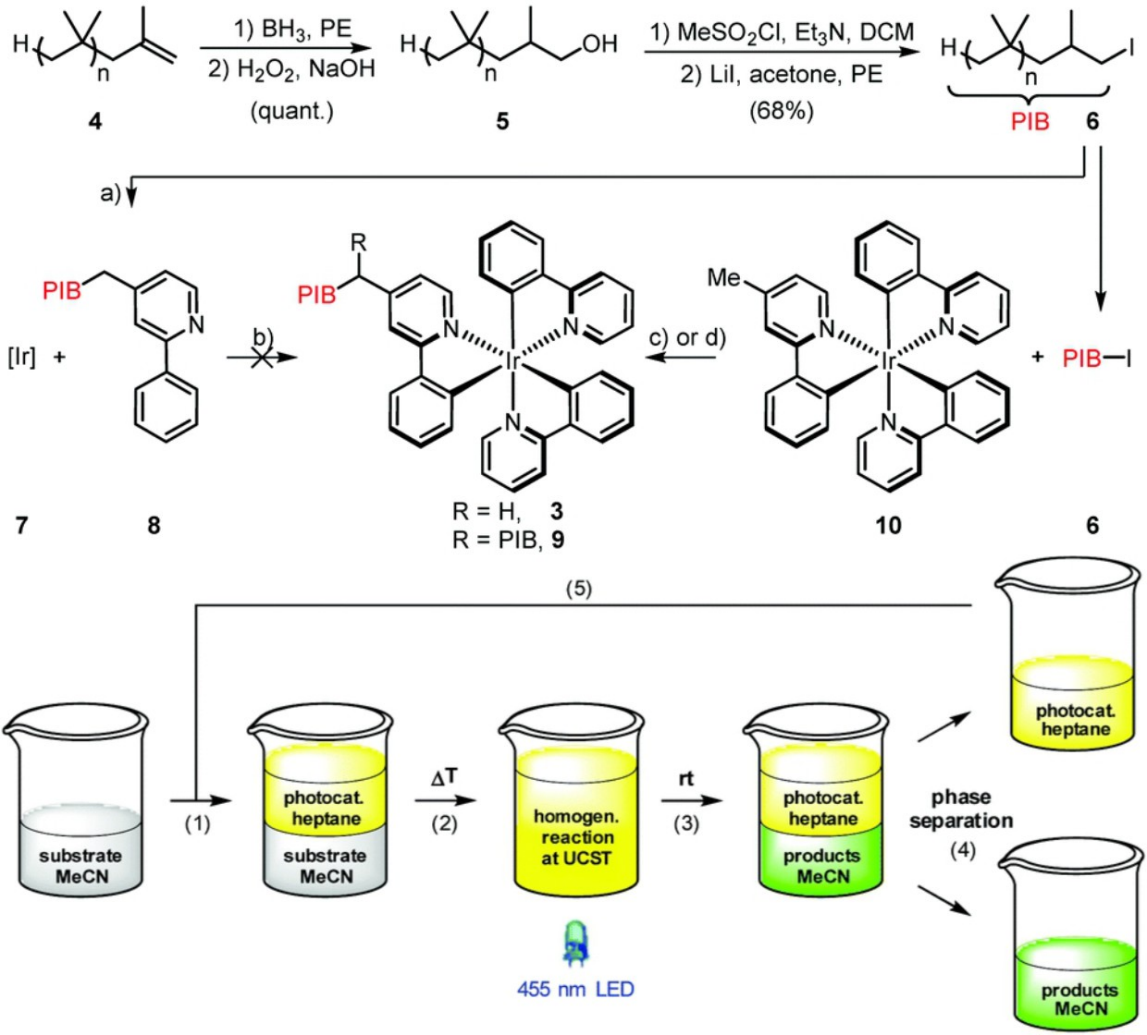
PIB coupling to Ir‐based photocatalyst. Method developed by Rackl et al.^[62]^ for photocatalysis in a biphasic system with easy separation of products and recyclability of photocatalyst. Figure reproduced from ref. [62].

The production of thermomorphic phase‐selective photocatalyst was further investigated by Rackl *et al*.^[62]^ They have developed single PIB polymer chain bonded iridium complexes that exhibit an almost identical optical property like its parent complex *fac*‐Ir(ppy)_3_ (Figure 4). Different from the aforementioned PIB coupling strategy, the authors synthesized the catalyst core structure prior to the attachment of the PIB chain. This PIB‐tagged photocatalyst was used in a biphasic thermomorphic system of heptane and acetonitrile that exhibits a UCST behavior. Initially, the substrates and reagents were dissolved in the acetonitrile at room temperature and to this a non‐miscible phase of heptane‐containing PIB‐tagged photocatalyst was added. This biphasic reaction mixture was heated exceeding the UCST to obtain a monophasic solution, allowing homogeneous photocatalysis. After the reaction was completed, the system was simply cooled to room temperature, returning to a two‐phase system that was separated to yield the product and recover the photocatalytic material. The catalyst was reused for at least 10 cycles, giving a constant high yield of product with comparable efficiency to the parent Ir photocatalyst. Moreover, the authors incorporated this system into a flow system to obtain continuous recycling of the photocatalyst. This flow system was applied in the photocatalytic *E*/*Z* isomerization of cinnamyl acetate, where high selectivity was obtained with an effective reduction of catalyst loading by a factor of 30.

TMSs based on PIB‐tagged metal complexes demonstrate a versatile strategy for the recovery and reuse of homogeneous transition metal catalysts. However, these polymer‐based systems display a molecular mass distribution without an accurate structure, which could lead to possible problems regarding solubility limitations and catalyst leaching.

More recently, long alkyl chains have been proved to be capable of generating a thermomorphic phase‐selective effect. A long alkyl chain (C_17_H_35_−) bound 2‐phenylpyridine Ir complex that possesses an accurate molecular weight has been designed and used for a wide scope of trifluoromethylation of aromatic and heterocyclic compounds with 43–82 % yield. This material showed negligible loss of activity after 5 cycles.^[63]^ Moreover, it should be addressed that these systems require high temperature (70 or 85 °C) in addition to light irradiation, which might be disadvantageous for temperature‐sensitive photochemical reactions.

## 3 pH‐Responsive Polymer Photocatalytic Systems

pH‐responsive polymers can also be employed to create responsive photocatalytic systems, in order to control their catalytic activity and facilitate their recovery by tuning the pH of the reaction medium. Commonly, pH‐responsive polymers are polyelectrolytes containing weakly ionizable groups, which can either accept or donate protons responding to the change of environmental pH.[^64^, ^65^, ^66^] At high pH conditions, polyacids are ionized, leading to a drastic change in hydrophilicity and electrostatic repulsion of the polymer chains. Examples are poly(acrylic acid) (PAA) and poly(methacrylic acid) (PMA).^[67]^ In contrast, polybases are protonated in neutral or acidic conditions, leading to an electrostatic repulsion and swelling of the polymeric materials. Examples are poly(dimethylamino ethylmethacylate) (PDMAEMA),^[68]^ poly(diisopropylamino ethylmethacylate) (PDIAEMA),^[69]^ and poly(4 or 2‐vinylpyridine) (P4VP or P2VP).^[70]^ This controllable swelling of these polymeric materials has been incorporated into photocatalytic systems, facilitating the dynamic control of the photoactivity and recovery of the photocatalytic materials. Ideally, light and reactants are free to reach the catalytic centers in the swollen state of the materials that facilitates efficient photocatalysis. Conversely, when the polymeric matrix is in a contracted state, the diffusion of the reactants and accessibility of reactive sites is hindered, resulting in a less efficient catalysis. The influence of pH variations on chemical reactions should be especially taken into consideration. For instance, a vast number of reactions^[71]^ prefer to take place under basic conditions, therefore an increase in pH value by itself favors the chemical reactions to proceed. Because of this complexity, the investigation of reaction kinetics variations in response to pH changes is difficult. Given the system displays a pH‐responsive swelling–shrinking behavior cannot provide comprehensive and precise information for the control of the reactions, complementary results to exclude the effect of pH variation on the reaction itself are expected. As a result, this pH‐induced phase transition is more commonly employed to facilitate the recovery of the photocatalytic materials.

### 3.1 Switchable Water Compatibility of Conjugated Polymers

Conjugated polymers consisting of fully π‐conjugated systems are appealing candidates for up‐scaling heterogeneous photocatalysis, because of their robustness, nontoxicity and high visible‐light activity. Considering the up‐scaling in more industrial applications, water, in particular, is considered as a green and economically friendly solvent for photocatalysis. However, the high photocatalytic performance of the conjugated polymer systems often has to compromise with the poor water solubility/dispersibility. Therefore, a great number of investigations have been carried out in order to improve their water compatibility.[^72^, ^73^, ^74^, ^75^, ^76^, ^77^, ^78^] Here, we would like to introduce two simple approaches through the addition of acid to improve the water compatibility of conjugated systems.

**Figure 5:**
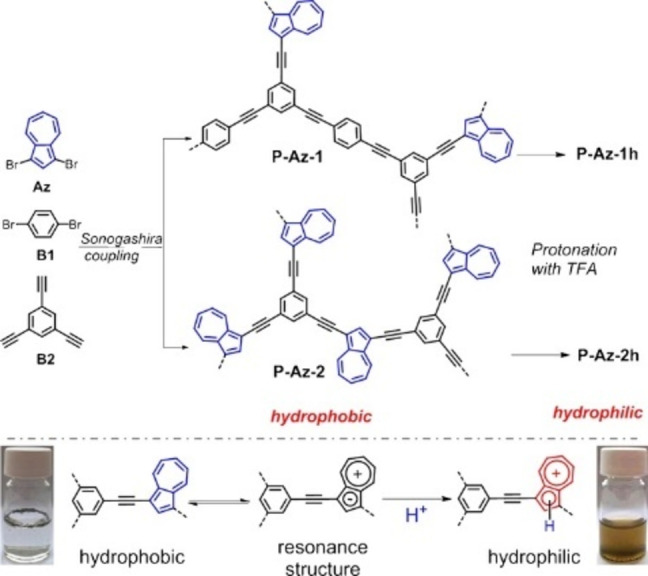
Synthetic pathway and modification method for hydrophilic, conjugated, microporous polyazulene networks. Reproduced from ref. [78].

A simple protonation approach of the pristine hydrophobic conjugated polymer networks has been reported by Zhang and co‐workers (Figure 5).^[78]^ Sonogashira cross‐coupling of 1,3‐dibromoazulene and 1,4‐dibromobenzene with 1,3,5‐triethynylbenzene as a cross‐linker was utilized for the synthesis of two conjugated microporous polymers (CMPs), which differ in the molar ratio of two monomers to cross‐linker. These CMPs contain azulene units that can be protonated in the presence of acid, resulting in the formation of a stable resonance aromatic six‐π‐electron tropylium cation. The authors took the advantage of this easy method and protonated these two CMPs using TFA, creating two hydrophilic CMPs that were well‐dispersed in water. These hydrophilic CMPs were employed for the photocatalytic reduction of Cr^VI^ pollutant to less toxic Cr^III^ and nearly full conversion was achieved after 1 h under the irradiation of a 23 W household energy‐saving lightbulb. Furthermore, in the presence of a metal cation Fe^III^ or Cu^II^ as a co‐catalyst, cascade catalytic cycles of Cr^VI^ reduction were accomplished without loss of activity after 5 runs, where the promoted electron in the conduction band of CMP is utilized to reduce either Fe^III^ or Cu^II^ to Fe^II^ or Cu^I^, respectively; this resultant reduced metal cation further reduces Cr^VI^, obtaining Cr^III^ and the pristine Fe^III^ or Cu^II^ cation. All these aspects demonstrate that these materials are promising water‐compatible heterogeneous photocatalysts. Notably, these two hydrophilic CMPs possess different valence bands and conduction bands, which gives the opportunity to choose a suitable photocatalyst according to the potential required for chemical reactions. It should also be mentioned that both the porosity and the pore volumes were reduced after protonation. This reduction was suspected to be caused by the trifluoroacetate as a counterion largely incorporated into the network to balance the tropylium cations. Additionally, the incorporation of the trifluoroacetate anions on the tropylium cations inside the protonated polymer network could also decrease the thermal stability of the material. At elevated temperatures, an increased mobility of trifluoroacetate anions is expected, leading to a reduction in the stability of the networks. Although the authors demonstrated efficient Cr^VI^ reduction using TFA‐protonated CMPs, the oxidation half reaction has not been specified.

**Figure 6:**
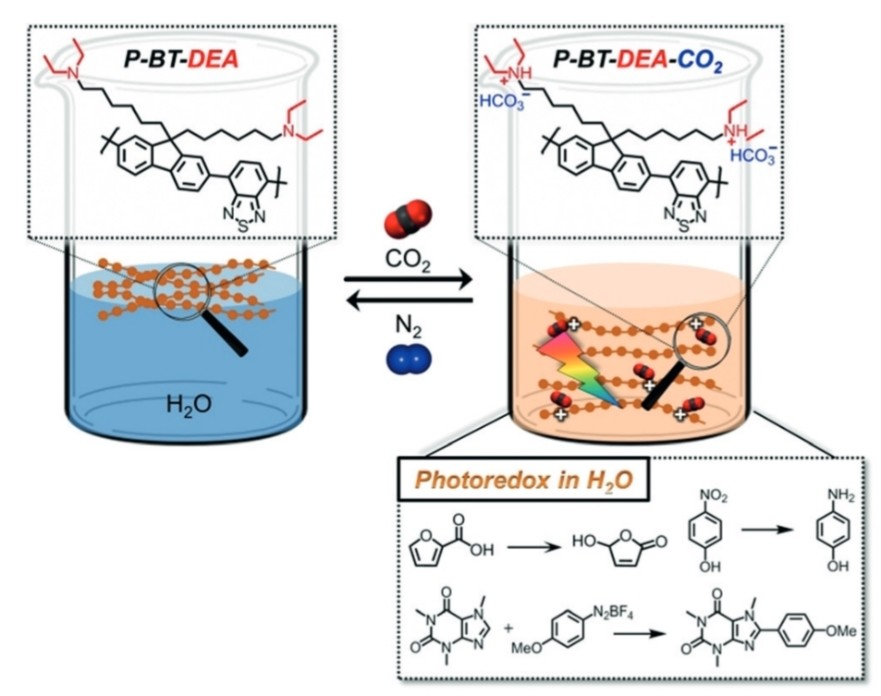
Schematic illustration of the switchable hydrophilicity of the conjugated polymer photocatalyst through CO_2_/N_2_ exchange, and its photocatalytic applications in water. Reproduced from ref. [79].

Further to the aforementioned system, CO_2_ has also been applied to reversibly tune the photoactivity of conjugated polymers as a special source of acid. Byun *et al*. modified the production of conjugated polymer photocatalyst with switchable hydrophilicity without compromising its recyclability (Figure 6).^[79]^ Here, a tertiary amine was implemented into the conjugated linear polymer through a post‐modification of hydrophobic P‐BT‐Br with diethylamine (DEA). The resulting P‐BT‐DEA was hydrophobic under neutral and basic conditions. In this complex, the tertiary amine acted as a proton acceptor upon being treated with CO_2_ gas, generating bicarbonate salts in aqueous solutions and facilitating a good polymer dispersion in water. The bicarbonate salts can be eliminated by treating the solution with N_2_ under mild conditions, leading to the precipitation and recycling of the hydrophobic P‐BT‐DEA. This CO_2_‐responsive photocatalyst was used for the effective dye degradation and photo‐redox reactions, including photo‐oxidation of 2‐furoic acid, photo‐reduction of 4‐nitrophenol (4‐NP), as well as the coupling of caffeine with aryldiazonium tetrafluoroborate. The efficient coupling of caffeine and aryldiazonium tetrafluoroborate as a model reaction of arylation of heteroarenes enables the direct production of biologically active compounds. Moreover, the PBT‐DEA‐CO_2_ could be reused for up to six 24 h cycles without a loss of conversion efficiency. This simple and reversible approach for switching the polymer solubility can be utilized as a versatile and powerful tool for functional photocatalyst synthesis in future investigations.

### 3.2 Switchable Aggregation and Disassembly

In the case of photodynamic therapy, pH variations across the tumor microenvironment can be used to selectively activate the photosensitizer at the tumor site, enabling a targeted treatment. For example, acid‐responsive AB2 Y‐shape metallo‐supramolecular micelles, containing iron‐tetraphenylporphyrins (Fe‐TPP) as a photosensitizer, have been fabricated by Wang *et al*. and used for photodynamic therapy.^[80]^ The micelles were formed through the metal–ligand interaction between Fe‐TPP and histidine. Histidine is an essential amino acid and it contains an imidazole group that can act as an acid‐liable ligand because of its weak base nature. Prior to the coordination process, histidine and photosensitizer were modified with hydrophobic PLA chains and hydrophilic PEG chains, respectively. At physiological pH (≈7.4), two histidine‐PLA chains were coordinated to one Fe‐TPP, creating Y‐shape supramolecular micelles (MSM), which can also be used for drug encapsulation. Once subjected to the tumor microenvironment (pH<6), imidazole groups were protonated, and thereafter, these metallo‐coordinated micelles were disassociated, revealing the photocatalytic sites and releasing the drugs. This disassembly was confirmed by dynamic light scattering (DLS) (increased hydrodynamic size and polydispersity index) and TEM (collapsed NPs). The photodynamic activity of MSM was characterized by singlet oxygen generation using 9, 10‐anthracenediyl‐bis(methylene) dimalonic acid (AMDA) as a trapping agent. However, the singlet oxygen generation of MSM has not been compared at pH 7.4 and pH 5.7. Moreover, at pH 7.4, the photobleaching rate of MSM was faster than that of the non‐responsive version (PDP). It is unclear what may cause this difference in these materials in terms of singlet oxygen generation at pH 7.4; however, the formation of PDP was through binding instead of coordination. In addition, binding could be expected instead of coordination. Furthermore, it could be expected that the photosensitizers are located at the interface of the hydrophilic PEG shell and hydrophobic PLA core in both cases, which may facilitate an identical accessibility of the oxygen and light towards the reactive centers. Therefore, the binding could possibly reduce the photoactivity of Fe‐TPP, conversely, the coordination approach demonstrates an efficient method for micelle formation without compromising Fe‐TPP activity. The acid‐liable photodynamic efficiency of MSM needs to be further addressed. Furthermore, the location and distribution of the photosensitizer should be taken into consideration for further investigations in order to have pH‐targeted activation for photodynamic therapy.

A more straightforward approach was reported by Chen and co‐workers,^[81]^ who utilized ATRP to produce a pH‐responsive amphiphilic block copolymer using a zinc tetraaminophthalocyanine derivative as the photocatalytic unit. Methacrylate‐modified zinc tetraaminophthalocyanine photocatalyst (ZnPc) monomers were copolymerized with pH‐responsive DIPAEMA monomers using a hydrophilic PEG−Br chain as initiator. At a pH value greater than 6, the PDIPAEMA block was deprotonated and highly hydrophobic, leading to a micelle formation through the self‐assembly of these amphiphilic polymer chains, whilst at a pH value less than 6, these micelles disassembled because the tertiary amine of the PDIPAEMA units became protonated. This protonation significantly increased hydrophilicity that led to a full solvation of the diblock copolymer and thereafter, the dissociation of the micelles. Here, the authors took advantage of this pH‐induced conformational change to switch the photocatalytic units “on” and “off”. The block copolymer photosensitizer was utilized to oxidize L‐tryptophan at pH 5.5 and 7.4, respectively, with an 8‐fold increase in yield observed at pH 5.5. This increased photoactivity at lower pH was attributed to the higher accessibility to the photosensitizer in the dissolved form compared with the micelles. Moreover, this pH‐sensitive photosensitizer is capable of responding to subtle pH changes in the range of tumor microenvironment, demonstrating a potential application in the field of photodynamic therapy.

**Figure 7:**
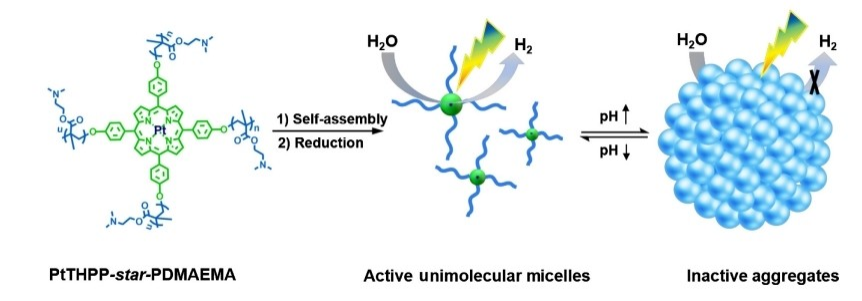
Schematic representation of switchable unimolecular micelles with single Pt atoms. The dispersed UMs are catalytically active, whereas the photocatalytic activity of the aggregated UMs is sharply reduced. Reproduced from ref. [51].

In addition to micelle formation and dissociation, the aggregation behavior of pH responsive systems has also been addressed. Zou *et al*.^[51]^ demonstrated the use of DMAEMA to produce pH‐responsive and recoverable photocatalysts containing a Pt porphyrin derivative (Figure 7). Four pH‐responsive PDMAEMA chains were extended from the hydrophobic Pt–porphyrin center, creating a star‐shaped unimolecular micelle (UM). At pH 2, the tertiary amine groups of PDMAEMA arms were fully protonated, resulting in UMs with excellent water dispersibility, unconstrained mass transfer, and high active site accessibility. When the pH was elevated above the p*K*_a_ (≈7.5) of PDMAEMA at pH 10, the PDMAEMA chains were deprotonated and became more hydrophobic, leading to the aggregation of UMs. This research also investigated the “on/off” switch of the photocatalytic activity and the recyclability of the photocatalyst, using the pH‐induced reversible dispersion/aggregation behavior of the UMs. The photocatalytic water splitting was studied at pH 2 and 10, respectively. A 12‐fold enhancement in H_2_ evolution was observed at pH 2, and the authors claimed that the formation of stable UM dispersions and high accessibility to the active catalytic sites are the reasons for the significant improvement in performance. However, the effect of pH on photocatalytic hydrogen evolution has not been clarified and further control experiments using non‐responsive photocatalyst for hydrogen evolution under both acidic and basic conditions may help support the claim.

### 3.3 Switchable Swelling and Collapse

Apart from the aforementioned temperature‐sensitive photocatalyst for PET‐RAFT polymerization, the implementation of pH‐responsive photocatalyst has emerged as an alternative powerful technique for spatiotemporal, composition, and sequence control of the polymer synthesis, in particular, under aqueous conditions. Huang *et al*.^[82]^ have prepared pH‐sensitive hollow microspheres immobilized with Zn‐based porphyrin (ZnTPP) as a heterogeneous photocatalyst. Briefly, a SiO_2_‐poly(glycidyl methacrylate‐*co*‐methacrylic acid) (PGMA‐*co*‐PMAA) core–shell structure was prepared prior to attaching ZnTPP moieties to the azide‐modified PGM‐N_3_ shell via alkyne–azide click chemistry. Followed by etching the SiO_2_ core using HF solution, pH‐sensitive hollow microspheres were obtained. The resulting microspheres self‐aggregated at a pH lower than 4.3, where the carboxylic groups, COOH, were protonated, whereas at neutral or basic conditions, the PMAA moieties became deprotonated, affording a homogeneous dispersion and porous shell structure due to increased hydrophilicity and charge repulsion. The authors investigated whether this structural and conformational change could be used to control the photoactivity of the photocatalytic units. These photocatalytic microspheres were utilized for the PET‐RAFT polymerization of *N*,*N*‐dimethylacrylamide (DMA) under three different pH values of 9.0, 7.0 and 5.0, respectively. A longer induction period and a slower propagation rate were observed at pH 5.0 in comparison to reactions performed at pH 7.0 and 9.0, which was attributed to the poor accessibility to the photocatalytic moieties compared with the complete dispersed and porous form. It should be noted that a reasonable propagation of polymer was still possible at pH 5.0, which may result from the distribution of the photocatalytic sites concentrated on the surface of the microspheres.

The use of an external trigger has been demonstrated to be a powerful and versatile approach to control photocatalytic reactions and recover the photocatalytic materials efficiently. So far, most of the photocatalytic control has been achieved through the modification of the physical environment of the photocatalytic sites; however, the modification of the nature of the photocatalytic center could also be used to induce switchability. These initial investigations into stimuli‐responsive photocatalysis have addressed their potential to produce a near limitless range of smart materials and can be translated into more industrially relevant applications.

## 4 Conclusion and Perspective

The combination of stimuli‐responsive systems with heterogeneous photocatalysts enables the production of a new class of catalyst featured by switchability, adaptability and excellent recyclability. To date, a variety of photoactive species have been implemented into a broad range of smart polymeric systems from linear polymers to gel matrix. The dynamic control over the photocatalytic performance of these materials can be generally achieved by regulating the accessibility of the photoactive sites according to the external environment. The switchable solubility/dispersibility of the stimuli‐responsive photocatalyst provides a precise temporal control regarding the on/off of the photocatalytic reactions. However, neither the regulation of the reaction rate in a continuous manner nor the quantity of the catalytic activity has been obtained. Moreover, no discussions regarding the changes in actual physicochemical processes upon the application of external triggers that occur in the photocatalysts, including photon absorption, exciton diffusion, and free charge generation, have been provided by the state‐of‐art investigations. These aspects are of great importance to understand the dynamic control and catalytic mechanism of the photocatalytic reactions truly generated from the macromolecular structural alternation upon applying stimulus.

Currently, temperature‐responsive photocatalytic polymers are still the most frequently and intensively investigated systems, especially for the systems based on PNIPAM. A number of new polymers with transition temperatures in a broad range from 20 to 50 °C^[83]^ have been developed recently, providing new possibilities to produce a palette of catalytic systems with different transition temperatures. Compared to temperature, fewer of the known smart photocatalytic systems are triggered by pH value changes, which should be further implemented and investigated. Especially, the orthogonal dynamic control over the photocatalytic reactions through the pH variation is desired. Furthermore, the production of switchable photocatalytic systems based on changing the nature of active centers still needs to be further investigated. For example, by directly altering photoactive sites in response to external stimuli, it is possible to tune the optical property of photocatalysts, which may be interesting for selective photocatalysis or cascade photocatalytic reactions. Additionally, the manipulation of the material's structure often leads to a contraction of the material, which may allow more efficient transfer of electrons or energy between synergistic species. To date this approach has still not been fully investigated.

In addition to heat and pH, many other triggers (i.e. salts, magnetic field, electric field, and etc.) could be implemented. For instance, the solubility of ionic liquid based polymers in water significantly depends on the counter ions of the polymers.^[84]^ Therefore, counter ion exchange can be utilized to switch the water solubility of ionic liquid based photocatalytic polymers, enabling the recovery of the photocatalyst and possible control of the photoactivity. Furthermore, the development of a flow device has opened the way in the direction for up‐scaling photocatalytic reactions with continuous product separation and catalyst recovery. However, most of these investigations are limited to the study of simple model reactions, including photooxygenation, dye degradation, and 4‐nitrophenol degradation, thus the production of more practically or technologically relevant compounds with high values (i.e. solar fuels or medicines) applying these materials would be extremely desirable. Stimuli‐responsive polymer photocatalysts are an exciting and sustainable class of hybrid materials with highly tunable potential, allowing the translation to more industrially relevant applications.

## Conflict of interest

The authors declare no conflict of interest.

## Biographical Information

*Rong Li received her Master of chemistry from University of Siegen in 2020. Currently she is working on her PhD at Max Planck Institute for Polymer Research under the supervision of Prof. Dr. Katharina Landfester. Her research interests focus on the synthesis of functional polymeric photocatalysts for photocatalytic and biomedical applications*.

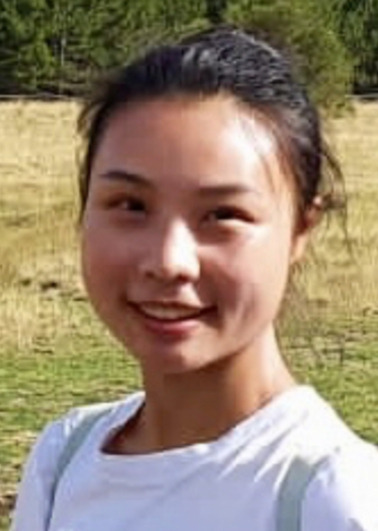

## Biographical Information

*Katharina Landfester received her doctoral degree in Physical Chemistry after working in 1995 at the MPI for Polymer Research (MPIP). After a postdoctoral stay at the Lehigh University (Bethlehem, PA), she worked at the MPI of Colloids and Interfaces in Golm leading the miniemulsion group. From 2003 to 2008, she was professor at the University of Ulm. She joined the Max Planck Society in 2008 as one of the directors of the MPIP. She was awarded the Reimund Stadler prize of the German Chemical Society and the prize of the Dr. Hermann Schnell Foundation, followed by the Bruno Werdelmann Lecturer in 2012 and the Bayer Lecturer in 2014. Her research focusses on creating functional colloids for new material and biomaterial applications*.

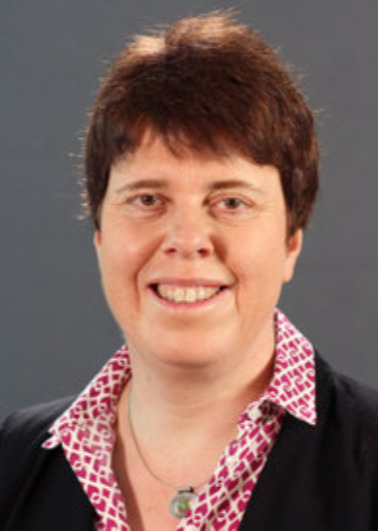

## Biographical Information

*Calum Ferguson undertook an integrated Masters in Chemistry at The University of Edinburgh. He then attained his PhD from the University of Leeds, UK, in 2018. After the completion of his doctoral studies, he joined the department of Physical Chemistry of Polymers at the Max Planck Institute for Polymer Research (MPIP), working with Prof. Katharina Landfester. Early 2020, he started as a research group leader at MPIP. In April 2022 he joined The University of Birmingham as a group leader in the O'Reilly group, whilst maintaining his group in Germany. His research interests include controlled radical polymer synthesis, photocatalytic classical polymers, organic small molecule photocatalysts, and the formation of bio‐mimicking polymers and colloids*.